# Optimization of a human milk–directed quantitative sIgA ELISA method substantiated by mass spectrometry

**DOI:** 10.1007/s00216-021-03468-4

**Published:** 2021-06-25

**Authors:** Kelly A. Dingess, Pauline van Dam, Jing Zhu, Marko Mank, Karen Knipping, Albert J.R. Heck, Bernd Stahl

**Affiliations:** 1 Biomolecular Mass Spectrometry and Proteomics, Bijvoet Center for Biomolecular Research and Utrecht Institute for Pharmaceutical Sciences, University of Utrecht, Padualaan 8, Utrecht, 3584 CH The Netherlands; 2 Netherlands Proteomics Center, Padualaan 8, Utrecht, 3584 CH The Netherlands; 3 Danone Nutricia Research, Uppsalalaan 12, Utrecht, 3584 CT The Netherlands; 4 Beijing Institute of Nutritional Resources, Beijing, 100069 China; 5 Chemical Biology & Drug Discovery, Utrecht Institute for Pharmaceutical Sciences, University of Utrecht, Universiteitsweg 99, Utrecht, 3584 CG The Netherlands

**Keywords:** Human milk, sIgA, ELISA, Mass spectrometry, Proteomics

## Abstract

Immunoglobulins are the primary protective products in human milk and are responsible for transferring maternal pathogen memory to the infant, providing protection by binding to recognized pathogens and inhibiting virulence. To better understand potentially protective/anti-infective compounds in human milk, the establishment of human milk–tailored analytical approaches is crucial, as most contemporary analytical methods have been optimized for plasma or serum. One of the most prominent immunoglobulins in human milk is secretory immunoglobulin A (sIgA), which may be relevant for the protection of breastfed infants from harmful pathogens. Advanced sIgA detection methods can help monitor the immune status and development of the mother-infant dyad. We therefore developed an enzyme-linked immunosorbent assay (ELISA) sIgA method for the quantitative analysis of IgA plus secretory component (SC), validated with sIgA standards and substantiated by mass spectrometry (MS)–based proteomics. A very strong correlation was observed between the MS-detected IgA1 and the human milk–specific sIgA ELISA (r = 0.82). Overall, the MS data indicate that the developed human milk sIgA ELISA does not differentiate between sIgA1 and sIgA2 and is, therefore, a reflection of total sIgA. Furthermore, our MS data and the human milk–derived sIgA ELISA data are better correlated than data derived from a standard serum IgA ELISA kit (relative to MS IgA1 r = 0.82 and r = 0.42, respectively). We therefore propose our human milk–specific sIgA ELISA as an ideal quantitative indicator of total sIgA with advantages over current serum IgA ELISA kits.

**Supplementary Information:**

The online version contains supplementary material available at 10.1007/s00216-021-03468-4.

## Introduction

Human milk is a highly complex biological matrix of fats, carbohydrates and proteins, cells, bacteria, and metabolites. However, the complexity of the human milk matrix is often overlooked and methods that have not been validated for human milk are used to assess its components. This has been shown to be problematic, not only in micronutrient analysis [1], but also when assessing human milk glycoproteins [2]. The constituents of the human milk matrix exclude plasma and serum–derived analytical methodologies from direct applications to human milk. Therefore, human milk–specific methods need to be established to characterize potentially protective components directed against pathogens, such as bacteria and viruses. Neonatal immunity is mediated by immunoglobulins (Igs) from human milk. There are varying Ig isotypes in human milk, with secretory IgA (sIgA) being by far the most abundant [3].

There are many structural differences between antibodies in serum and milk. Moreover, human Igs are distinctly different from those of other mammalian species, as humans have two subclasses of IgA: IgA1 and IgA2. These IgA subclasses are structurally different throughout the body. Serum IgA1 and IgA2 are predominantly monomeric; sIgAs secreted at mucosal epithelial cell surfaces are trimeric or tetrameric; and the two isotypes sIgA1 and sIgA2 present in human milk are predominantly dimeric [4]. Due to these various structural differences, sIgA is an inherently more complex Ig than others, like IgG. This is largely attributed to the joining chain (JC) and secretory component (SC) associated with IgA forming sIgA, which are lacking in IgG. Recently, refined structural models of IgA were published, suggesting a mechanism in which the JC component of sIgA drives higher-order oligomerization [5]. This is a unique feature of IgA and IgM, as the only two Igs that contain the JC. Without this JC component, IgG remains a monomer throughout the body and does not form higher-order oligomers. This structural difference contributes to differing functions of sIgA and IgG, and it is generally accepted that higher-order oligomeric states of sIgA display better pathogen-neutralizing capacities than monomers or dimers [5].

Serum Igs are either natural or antigen induced [6] and levels are controlled by feedback mechanisms [7, 8]. Human milk Igs are thought to be both natural and antigen induced and provide a broad spectrum of protection to the infant. Therefore, they are a reflection of maternal antigenic stimulated immunity [9]. The memory of pathogens faced by the mother is carried by Igs, including sIgAs, providing the same protection to the infant by binding to recognized pathogens and inhibiting their ability to cause infection [10]. Importantly, sIgA and free SC resist digestion in the infant’s stomach [11]. This is an important evolutionary adaptation, as the immature intestinal mucosa of the infant is overly sensitive to infection due to overexpressed inflammatory genes and under-expressed negative feedback regulator genes [12]. Human milk helps regulate this immunologic balance in infants, not only by reducing pathogen exposure and prevention of infections, but also by modulating the immune response to minimize inflammatory events [13].

To accurately characterize sIgA in human milk, we aimed to optimize and validate a human milk–specific sIgA ELISA method, ensuring more insightful information regarding both maternal and infant health. Our newly developed sIgA ELISA method was substantiated by cross-correlating data derived from mass spectrometry (MS) and a commercially available serum IgA ELISA kit. Overall, we provide here a validated method that is able to quantitatively determine sIgA in human milk with high accuracy and at relatively high throughput.

## Methods

### Study design

To validate our ELISA-based method, we performed replicated measurements against a standard curve, and tested the effects of freeze/thaw cycles. To further support our findings, our human milk sIgA ELISA was tested against a commercially available serum IgA kit and all results were correlated with MS-derived data. Comparative analysis was also done with IgG to show the inherent differences in Ig complexity and the considerations needed for developing human milk–specific methodologies.

### Samples

Details of subjects and the human milk samples used in this study as well as label-free quantification (LFQ) of shotgun proteomics methodologies have been extensively described previously [14, 15]. Briefly, human milk samples were collected from two individual donors across weeks 1, 2, 3, 4, 6, 8, 10, 12, and 16 of lactation under standardized conditions [16]. These same human milk samples were used to develop the current ELISA method, so that the MS results from the previous study could be used to support the specificity of the ELISA method, detailed below, for sIgA.

#### Whole-milk shotgun proteome analysis

Whole-milk proteins were extracted, reduced, alkylated, and then digested overnight, as previously reported [15]. Tryptic peptides were separated and analyzed using liquid chromatography (LC)-MS/MS; an Agilent 1290 Infinity HPLC system (Agilent Technologies, Waldbronn, Germany) was coupled to a Q Exactive Plus hybrid quadrupole-Orbitrap mass spectrometer (Thermo Fisher Scientific, Bremen, Germany). The MS was operated in data-dependent acquisition mode, and high-energy collision dissociation (HCD) was used for MS/MS fragmentation. Raw LC-MS/MS data were searched with Proteome Discoverer (PD) (version 2.2, Thermo Scientific) using the Mascot search engine (version 2.6.1) against a UniProt Swiss-Prot database [17]: *Homo sapiens* (canonical and isoform) (December 2018, 20,417 entries) and filtered by 1% false discovery rate (FDR).

Protein concentration from shotgun, label-free quantification (LFQ) MS was derived from quantified amino acid analysis. This has previously been reported [15]. Briefly, protein pellets were precipitated from whole milk samples with 20% TCA (1:1 v/v); this extraction was then used for amino acid (AA) hydrolysis to determine total AA content to derive individual protein quantification. Total AA hydrolysis was achieved by addition of hydrochloric acid (Sigma-Aldrich) 6 N, followed by heating for 20 h at 110 °C. Neutralized, washed samples were analyzed by LC-MS performed on a Dionex Ultimate 3000 autosampler and pump (Thermo Scientific) coupled on-line to a Q Exactive mass spectrometer (Thermo Scientific), and separated by a Sequant ZIC-pHILIC column (2.1 × 150 mm, 5 μm, guard column 2.1 × 20 mm, 5 μm; Merck). The MS operated in polarity-switching mode. AAs were identified on the basis of exact mass within 5 ppm and further validated by retention times of standards. Quantification was based on peak area using LCquan software (Thermo Scientific).

Protein intensity, from LFQ data, was determined by the average intensity of all unique peptides. A protein quantitation index (PQI) was calculated using the mean intensity of unique peptides direct proportionality between PQI and protein abundance. Briefly, the total AA concentration was used to estimate the abundance of each protein by calculating the proportion of the mean intensity of unique peptides for each protein to the total unique peptide intensity. Full descriptions and the equations used are described in detail [15].

#### Whole milk targeted proteomics for IgA1

Parallel reaction monitoring (PRM) was performed for IgA1 to confirm LFQ protein concentrations. PRM peptides were chosen based on general rules for targeted proteomics [18]. For the selected peptides, stable isotope-labeled standards terminated with C-terminal heavy Arg/Lys were purchased from JPT Innovative Peptide Solutions for the heavy constant regions of IgA1: TFTCTAAYPES**K** and TPLTATLS**K**. Crude peptides had an assumed concentration of 14 nmol/well and were diluted to 1e5 fmol/μL for analysis. MS/MS analysis and database searching have been previously described [15]. Briefly, tryptic digested peptides with spiked heavy labeled peptides and Pierce™ Peptide Retention Time Calibration Mixture (PRTC) were analyzed by LC-MS/MS using an UltiMate 3000 HPLC (Thermo Fisher Scientific, Bremen, Germany) coupled online to a Q Exactive High Field X quadrupole-Orbitrap mass spectrometer (Thermo Fisher Scientific, Bremen, Germany).

For determining concentrations of the IgA1 peptides, increasing concentrations of the stable isotope-labeled peptide mixture were spiked into milk samples, generating a standard curve ranging from approximately 1 to 50 fmol. Summing extracted peak areas of non-interfered fragment ions, per precursor ion, were used for calculating ratios of light to heavy in the pair of each target peptide and its stable isotope standard. Peptide amounts were generated by ratio of light to heavy multiplying amount of stable isotope-labeled peptide. Peptides were then further derived to molar concentrations and microgram-per-milliliter concentrations by multiplying the derived ratio by the amount of heavy peptide added (fmol) and by the molecular weight of the peptide (Da), consecutively.

### Commercially available sandwich ELISAs

Complete kits were purchased for 2 Igs, IgA (Affymetrix eBioscience, BMS2096) and IgG (Affymetrix eBioscience, BMS2091). The kits were applied to human milk samples following the manufacturer’s instructions as recommended for serum. A 7-point standard curve enabled quantitation of individual Igs respectively. Both ELISA kits were validated for serum or plasma by the manufacturers. Preliminary testing was done to determine dilution factors suitable for human milk samples. All samples were defatted by centrifugation (1500g × 20 min × 4 °C), removing the bottom aqueous layer to a new tube. Samples were skimmed two times prior to analysis and plated in duplicate with dilutions of 1:10,000 and 1:1000 for IgA and IgG respectively. Dilutions were chosen based on derived LFQ concentrations for each individual Ig and the concentration range of the respective ELISA kit.

### Secretory IgA (sIgA) ELISA

The quantitative sIgA ELISA assay utilizes the two-site “sandwich” technique with two selected antibodies (monoclonal and polyclonal) that bind to human sIgA. Wells of a microtiter plate (Corning, Merck, Darmstadt, Germany) were coated with a capture antibody mouse α-human secretory component IgA (clone GA-1; Sigma-Aldrich) 1 μg/mL in carbonate-bicarbonate buffer (Sigma-Aldrich) and incubated at 4 °C overnight. Wells were washed 4× with 300 μL PBS (Fisher reagents) + 0.05% Tween-20 (Merck) and blocked with demineralized water +1% fish gelatin (Sigma-Aldrich) for 2 h at room temperature (RT) under continuous shaking. Assay standards were prepared with sIgA from Human colostrum (Sigma) with a range of 10–640 ng/mL in LowCross buffer (Candor Bioscience GmbH). Wells were washed as described previously, 100 μL assay standards and 10,000× prediluted skimmed human milk samples in LowCross buffer were added in duplicate to the appropriate wells and incubated for 2 h at RT under continuous shaking. Human milk samples were defatted as mentioned above. Wells were washed and 100 μL Biotin Mouse α-Human IgA1/IgA2 antibody (clone G20–359; BD Pharmingen) 0.25 μg/mL diluted in LowCross buffer was added to each well and incubated for 1 h at RT under continuous shaking. Wells were washed and 100 μL Poly-streptavidin-HRP (Sanquin) 15,000× diluted in LowCross buffer was added to each well and incubated for 30 min at RT and protected from direct light. Wells were washed; 100 μL Tetra methyl-benzidine (TMB; Thermo Scientific) substrate was added to each well and incubated for 5 min at RT and protected from direct light. The reaction was stopped by adding 100 μL of sulfuric acid solution 2 N (H_2_SO_4_; Merck). The optical density (OD) was measured at 450 nm and the OD is directly proportional to the concentration of sIgA. A dose-response curve of the OD versus concentration was generated, using the values obtained from the assay standards, and sIgA concentrations in the samples were calculated using the standard curve; see Supplementary Information (ESM) Table S1 and Fig. S1.

### Statistical analysis

The statistical analysis, Pearson correlations, and all figures were generated with R version 3.4.2, using ggplot2 (version 2.2.1). Pearson correlations were considered very strong with r ≥ 0.8, strong 0.6 < r < 0.79, moderate 0.4 < r < 0.59, weak 0.2 < r < 0.39, and very weak r < 0.19 [19]. Pearson correlations were preformed to compare MS and ELISA methods. Validation statistical calculations for the developed ELISA were performed using GraphPad Prism 8.

## Results and discussion

**Fig. 1:**
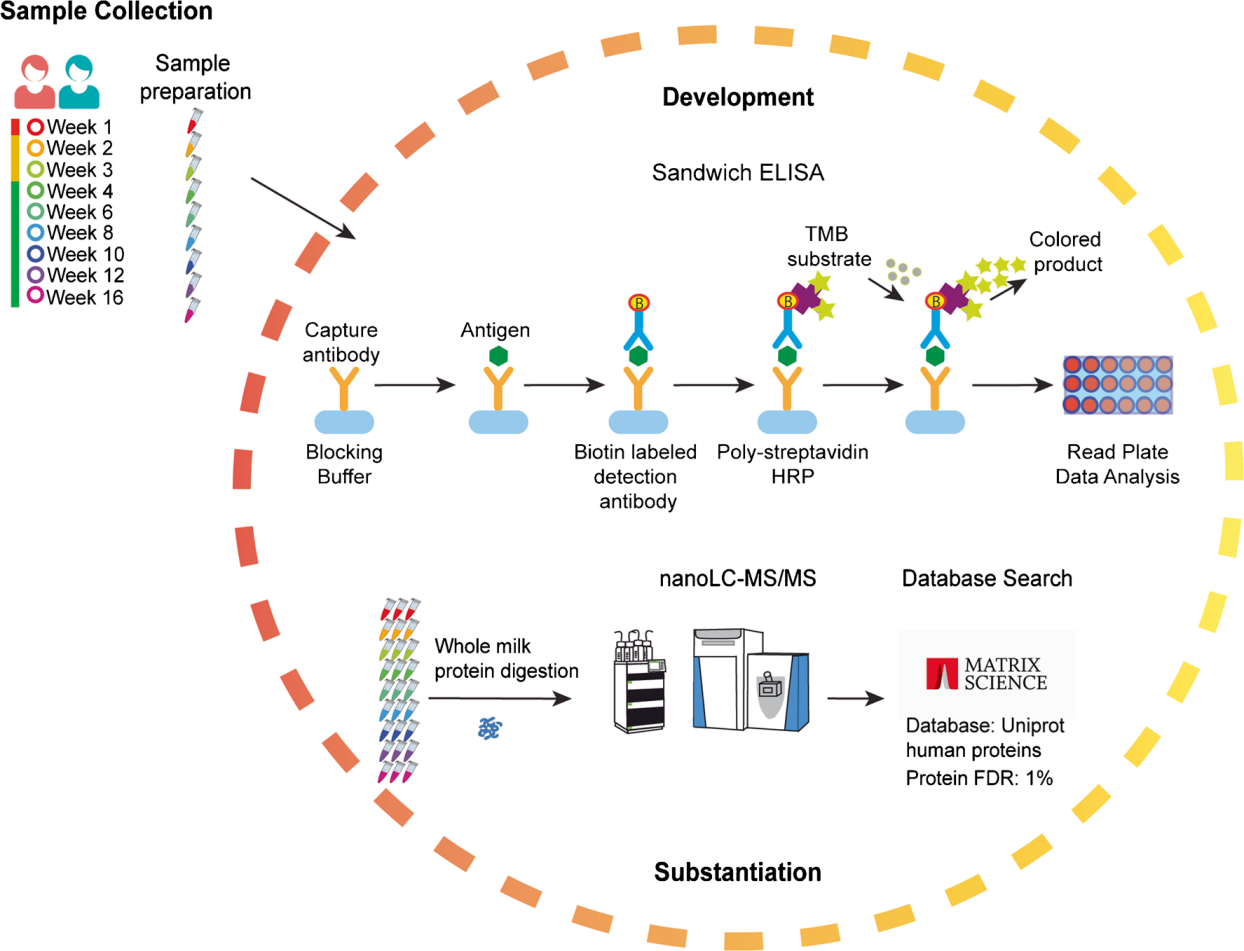
Workflow for the development of a human milk–specific sIgA ELISA substantiated by MS. Samples from two individual donors were used in the development and validation of a sandwich ELISA method for sIgA in human milk. Samples were analyzed in duplicate using a standard curve to determine concentration. MS methods were used to substantiate the newly developed sIgA ELISA method. Absolute concentrations from both methods were compared by plotting side by side, and by Pearson correlation

**Fig. 2:**
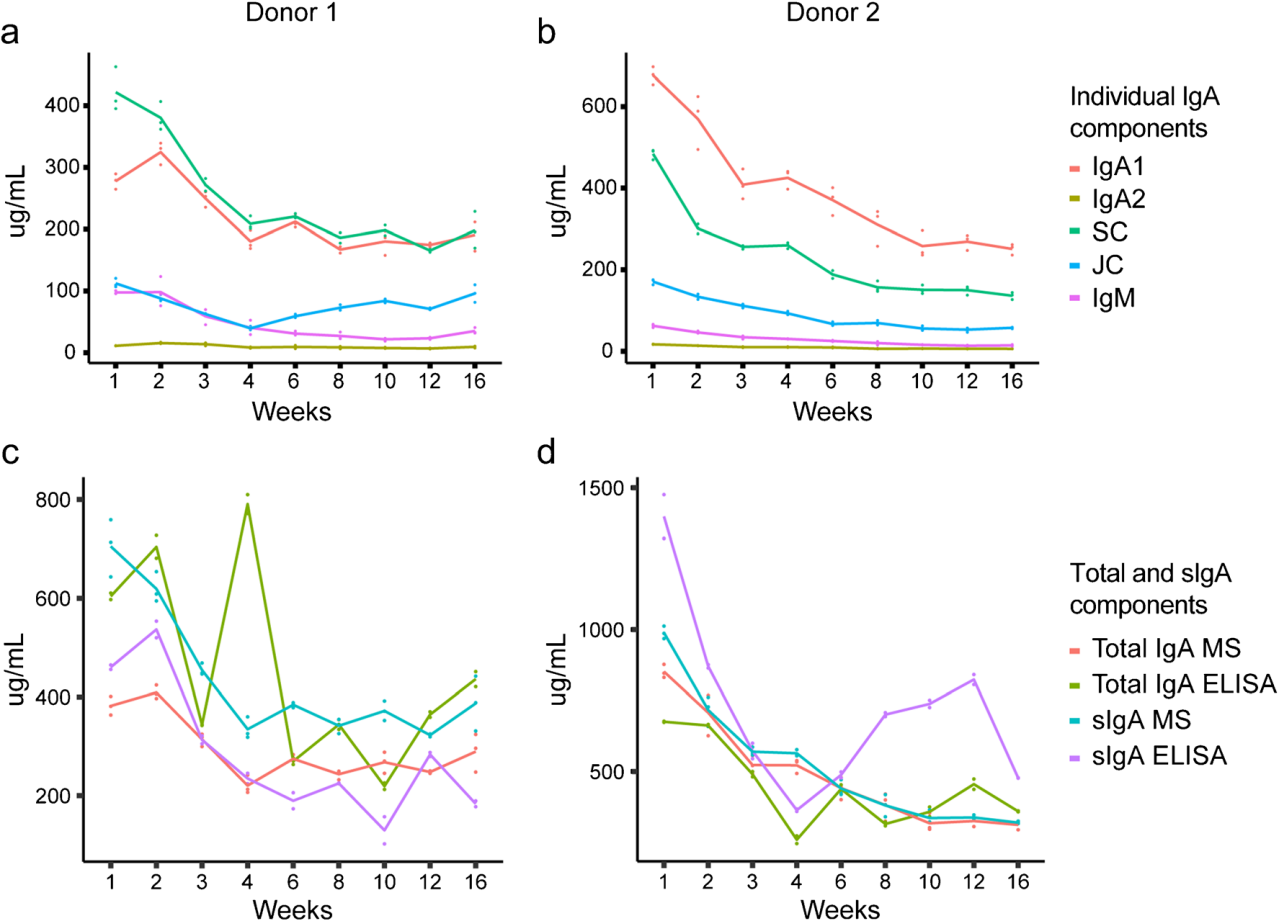
Comparative concentrations (μg/mL) of IgA components from MS and ELISA across lactation for individual donors. Trends in concentration of individual IgA1 and IgA2, total IgA and sIgA components from MS and ELISA data depicting the two donors from weeks 1 to 16. **a**, **b** Individual IgA components from MS data, including IgA1, IgA2, SC, JC, and IgM from donor one and two, respectively. **c**, **d** Total IgA and sIgA from MS, serum ELISA kit, and the human milk sIgA–specific ELISA for donors one and two, respectively. Data points indicate the values of each technical replicate; lines are linked by the median of the data points in each week for each donor

**Fig. 3:**
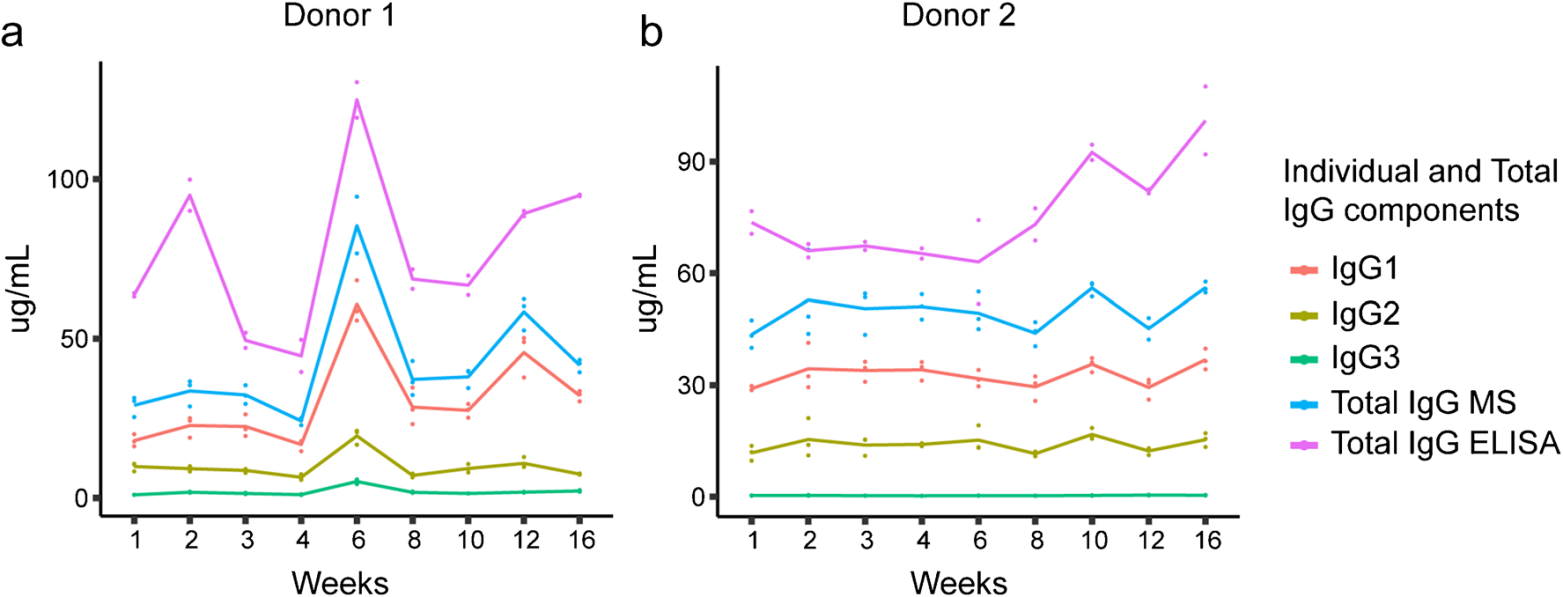
Comparative concentrations (μg/mL) of IgG from MS and ELISA across lactation for individual donors. Trends in concentration of MS and ELISA data from weeks 1 to 16 in donors one and two, respectively. IgG1, IgG2, IgG3, and total IgG from MS and the serum ELISA kit are depicted. Data points indicate the values of each technical replicate; lines are linked by the median of the data points in each week for each donor

We sought to establish a human milk–specific ELISA method for the quantitative analysis of sIgA. For this, two longitudinal series of human milk samples from two different donors were collected from weeks 1 to 16 postpartum and comparatively analyzed. A comparison of our new sIgA-specific ELISA method for human milk with a commercially available serum ELISA kit for IgA and MS of the heavy constant region of IgA was done. Additionally, we were able to compare IgA and IgG across the differing methodologies. We could show that different antibody classes, IgA or IgG, and sample types, milk or serum, are critical factors when developing new quantitative methods to characterize Igs, following the workflow depicted in Fig. 1. Results for the quantitative comparison of different Igs across different methodologies are depicted for IgA and IgG in Figs. 2 and 3, respectively.

### Development and validation of a human milk–specific sandwich sIgA ELISA

Since human milk is a complex matrix, a validation of the sandwich sIgA ELISA was done for human milk. Validation was performed according to the guidelines from the European Medicines Agency (EMA, Guideline on bioanalytical method validation EMEA/CHMP/EWP/192217/2009 Rev. 1 Corr. 2** 21 July 2011) to ensure appropriately validated methods with defined acceptance criteria [20, 21]. The assay was already fully validated for human serum and fecal samples. In addition, we used 2 different human milk samples to investigate suitability of the assay format for human milk. For the intra-assay variation (criteria coefficient of variation (CV) % of duplicates ≤ 15%, except for the lower limit of quantification (LLOQ), CV% should be ≤ 20%), the human milk samples were within the acceptable range (1.4–4.9%) [20, 21], ESM Fig. S1a. The inter-assay variation (2 human milk samples tested in 3 separate assays on 3 different days performed by 2 persons), with the same criteria as intra-assay variation, showed higher CV% variation (22.6–29.8%), ESM Fig. S1a. One possible reason for high intra-assay variation could be the rapid colorimetric development of the assay (TMB substrate, quenched by sulfuric acid solution 2 N). As this is highly time dependent, this is one of the most critical steps of the assay, and if preformed differently can result in slight differences between different days. Therefore, it is advisable to compare samples in the same assay performed by the same individual, or a control sample should be added when measuring on different days and different individuals to control for the variation. The sandwich ELISA analysis described here was performed in the same assay by one individual to avoid inter-assay variability.

Two aspects to this newly developed method, which make it specialized to human milk, are the assay standard sIgA from human colostrum (Sigma) and the use of LowCross buffer (Candor Bioscience GmbH). These two aspects allow for this ELISA method to measure the concentration of human milk sIgA against a human milk sIgA standard curve. This is advantageous to other ELISA kits developed for serum or saliva, which measure often against monoclonal antibodies that may or may not be similar to those found back in human milk. The advantage of using the LowCross buffer is that it minimizes the effect of the complex milk matrix. As other biofluids like serum and saliva do not contain matrixes complicated by components like highly abundant fatty acids and oligosaccharides, therefore, the buffers used in these ELISA kits do not account for the milk matrix. Combining these two aspects of our method makes it unique relative to commercially available kits, and specific to human milk sIgA.

Further, we assessed the cross reactivity of the IgA antibodies used in our assay. The monoclonal anti-human secretory component (Clone GA-1) is immunospecific for secretory human IgA and the free SC, and does not react with human IgG, IgM, or IgE. The Biotin Mouse anti-human IgA1/IgA2 (Clone G20–359) is specific for human IgA1 and IgA2 and does not react with other Ig isotypes. Two different lot numbers of the antibodies were tested and showed no effect on results; data not shown. The upper limit of quantitation (ULOQ) is determined by the highest standard (640 ng/mL). The highest standard was measured with an accuracy of 98.1% and a precision of 2.4%. The lowest limit of quantitation (LLOQ) is the lowest concentration of analyte in a sample which can be quantified reliably, with acceptable accuracy and precision. The LLOQ is considered being the lowest calibration standard (10.0 ng/mL). The lowest calibration standard (0.296) is at least 5× the signal of the blank (0.056). The observed detection limit (LOD) of 10 ng/mL was defined by the lowest sIgA concentration that is statistically distinguishable from the background. The significant difference between the OD of the blank against the OD of the lowest standard is determined with a student’s t test. The OD of the lowest standard differed significantly (*p* = 0.017) from the OD of the blank. The lowest calibration standard was measured with an accuracy of 94.1% (%recovery), a precision of 5.1% (%CV), and an OD with a CV of 35.7%, ESM Fig. S1b and S1c.

To investigate the linearity of the assay for human milk, a sample dilution series (2500×, 5000×, 10,000×, and 20,000×) was performed and showed linearity, parallel in slope compared to the standard line, ESM Table S3 and Fig. S2a. The matrix effect was studied by performing a matrix spiking with a known concentration of sIgA and is acceptable when spike recovery is between 50 and 150% [20, 21]. Samples were diluted 10,000× and 20,000× and spiked with 80 ng/mL of sIgA standard. The spike recovery of sIgA in human milk samples was between 60.6 and 104.9%, ESM Table S4. The stability of inter-assay comparability for sIgA in human milk samples was tested after 1, 2, and 3 freeze/thaw cycles, and showed to be stable after 3 freeze/thaw cycles (CV < 10%), ESM Table S2 and Fig. S2b. While these differences in concentration were not significantly different between freeze/thaw cycles for inter-assay variability, it is advised to always measure samples that have undergone the same freeze/thaw exposure to ensure accurate sample concentration assessment between samples. It has been well documented that the concentration and functional activity of sIgAs are affected by heat-treated pasteurization [22, 23]. Moreover, it is known that in general human milk proteins are susceptible to degradation when stored at higher temperatures and that this is only avoided when samples are stored at − 80 °C [24]. To avoid the influence of temperature effects, all samples in this study were stored at − 80 °C until analysis and all were compared under the same freeze/thaw cycle conditions; no samples were analyzed after three freeze/thaw cycles.

### Comparative analysis of Igs by MS and ELISA

In human milk, IgAs are typically present in secretory forms, in which the dimeric IgA1 and IgA2 are covalently bonded to a JC and SC (individual plots in Fig. 2, molarity in Table 1). The SC results from the endoproteolytic cleavage of the polymeric immunoglobulin receptor (pIgR) [25]. SC and JC were also quantified by MS (Fig. 2). These are important components to consider when developing a new ELISA for sIgA as they are all part of the protein complex and differences in association may affect binding affinity.

**Table 1:** MS-determined molarity of IgAs, IgM, JC, and SC

	Measured mean (g/mol) across replicates^1^					Assumed molarity by oligomeric state			Relative molarity of assumed to measured	
	IgA1	IgA2	IgM	JC	SC	JC for total IgM^2^	JC for total IgA^3^	SC for total IgA^3^	% JC to IgA^3^	% SC to IgA^3^
Donor 1 Week 1	7.38E−06	2.30E−07	1.98E−06	6.21E−06	5.07E−06	3.95E−07	3.80E−06	3.80E−06	61	75
Donor 1 Week 2	8.62E−06	3.20E−07	1.99E−06	4.86E−06	4.57E−06	3.98E−07	4.47E−06	4.47E−06	92	98
Donor 1 Week 3	6.63E−06	2.81E−07	1.19E−06	3.46E−06	3.26E−06	2.39E−07	3.46E−06	3.46E−06	100	106
Donor 1 Week 4	4.79E−06	2.13E−07	8.17E−07	2.19E−06	2.51E−06	1.63E−07	2.50E−06	2.50E−06	114	100
Donor 1 Week 6	5.63E−06	1.57E−07	6.26E−07	3.27E−06	2.65E−06	1.25E−07	2.90E−06	2.90E−06	89	109
Donor 1 Week 8	4.44E−06	1.62E−07	5.50E−07	4.03E−06	2.24E−06	1.10E−07	2.30E−06	2.30E−06	57	103
Donor 1 Week 10	4.79E−06	1.53E−07	4.40E−07	4.64E−06	2.38E−06	8.79E−08	2.47E−06	2.47E−06	53	104
Donor 1 Week 12	4.64E−06	1.72E−07	4.74E−07	3.92E−06	1.99E−06	9.48E−08	2.41E−06	2.41E−06	61	121
Donor 1 Week 16	5.07E−06	3.01E−07	7.13E−07	5.31E−06	2.38E−06	1.43E−07	2.68E−06	2.68E−06	51	113
Donor 2 Week 1	1.80E−05	3.04E−07	1.26E−06	9.44E−06	5.81E−06	2.52E−07	9.13E−06	9.13E−06	97	157
Donor 2 Week 2	1.51E−05	2.23E−07	9.33E−07	7.39E−06	3.61E−06	1.87E−07	7.67E−06	7.67E−06	104	212
Donor 2 Week 3	1.08E−05	2.05E−07	6.98E−07	6.16E−06	3.08E−06	1.40E−07	5.52E−06	5.52E−06	90	180
Donor 2 Week 4	1.13E−05	1.93E−07	6.11E−07	5.14E−06	3.12E−06	1.22E−07	5.74E−06	5.74E−06	112	184
Donor 2 Week 6	9.84E−06	1.58E−07	5.07E−07	3.69E−06	2.26E−06	1.01E−07	5.00E−06	5.00E−06	135	221
Donor 2 Week 8	8.24E−06	1.25E−07	4.05E−07	3.83E−06	1.88E−06	8.10E−08	4.18E−06	4.18E−06	109	222
Donor 2 Week 10	6.85E−06	1.25E−07	3.16E−07	3.08E−06	1.81E−06	6.33E−08	3.49E−06	3.49E−06	114	192
Donor 2 Week 12	7.14E−06	1.25E−07	2.74E−07	2.92E−06	1.80E−06	5.49E−08	3.63E−06	3.63E−06	126	203
Donor 2 Week 16	6.67E−06	1.51E−07	2.89E−07	3.18E−06	1.64E−06	5.78E−08	3.41E−06	3.41E−06	107	209

^1^Molar concentrations derived from measured concentration values as the mean of three MS replicates

^2^IgM was assumed to be a pentamer

^3^The sum of IgA1 and IgA2

Using our LFQ proteomics approach, we can distinguish between the constant heavy chains of various Igs and use this information to estimate the concentration of different Igs in human milk. Protein concentrations are determined by a protein quantitation index (PQI) and further calculated to milligrams-per-milliliter concentrations from experimentally determined amino acid concentrations per individual sample, described in detail in Zhu et al. [15]. With this approach, it is possible to quantify different subclasses of Igs across lactation, including IgAs (IgA1 and IgA2) and IgGs (IgG1, IgG2, and IgG3). In the current analysis, the molar concentrations (g/mol) are determined from the calculated concentration (mg/mL) and Ig molar masses of protein backbone from Uniprot. As IgA was the protein of interest in our assay development, we confirmed LFQ concentrations with a targeted quantitative validation by parallel reaction monitoring (PRM). PRM uses high-resolution and high-precision MS, allowing for absolute protein quantification by monitoring spiked heavy isotopically labeled peptides to the endogenous peptide measured. We could validate that our LFQ MS method accurately measures the concentration of IgA1, for the heavy constant region of the protein (ESM Fig. S3).

For calculating sIgA from LFQ MS data, an assumption of 2 mol of total IgA, the sum of IgA1 and IgA2, plus 1 mol of JC and 1 mol of SC, was used. To make this assumption, the molar concentration of total IgA was divided by 2, as we assume the majority of IgA is dimeric. The association of JC with IgA was then determined after accounting for the amount of JC assumed to be associated with IgM, where 5 mol of IgM is bound to 1 mol of JC. Likewise, for IgM, if we assume the majority is pentameric, the molar concentration was divided by 5. These values are described in Table 1, wherein MS data reveals that differing stages of lactation and individual donor impact the expected to determined outcomes for all components of sIgA. In these assumptions, percentages of JC or SC to IgA > 100% indicate free JC or SC, or at least JC and SC not bound to IgA, and percentages < 100% indicate higher-order oligomers of IgA than the assumed dimer. For instance, in donor two, in all time points, we observed between 90 and 135% of JC associated with total IgA. This is indicative of the majority of sIgAs from donor two being dimeric, with possibly some free JC as the JC to IgA ratio exceeds 100% utilization. Whereas, in donor one, the association of JC to total IgA was observed to be 51–104%, indicating that possibly higher-order oligomeric IgAs were making up a greater percentage of samples at differing lactational stages as not all the JC was being used. Another check was to investigate how much SC was being used by total IgA, and again each donor was different. Donors one and two ranged between 75–121% and 157–222% respectively, indicating that there was free SC, or non-IgA-associated SC, present in both donors but to a much greater extent in donor two. Unfortunately, with LFQ MS data, it is not possible to distinguish free SC from IgA- or IgM-bound SC.

Determining in parallel different Igs and their subclasses in one experiment is an advantage of LFQ MS vs ELISA. Additionally, LFQ MS is an accepted method for identifying and quantifying proteins without having knowledge of what or how much of a given protein is present in a sample prior to analysis, which is necessary for PRM MS experiments. Moreover, PRM experiments are more costly than LFQ because of the need for synthetic heavy isotopically labeled peptides. Identifying differing Igs and their subclasses is often only possible by applying different ELISA methods specific for the target of interest or by setting up a multiplex assay. Currently available multiplex assays for IgA and individual subclass ELISA kits are only available for serum for monomeric IgA. More often, the detection of IgA or IgG by ELISA is done as total Ig and could be due to binding affinity with any of the IgA or IgG subclasses. For this reason, a comparison of MS and ELISA data can give insight into the specificity and accuracy of our developed human milk–specific sIgA ELISA method.

**Fig. 4:**
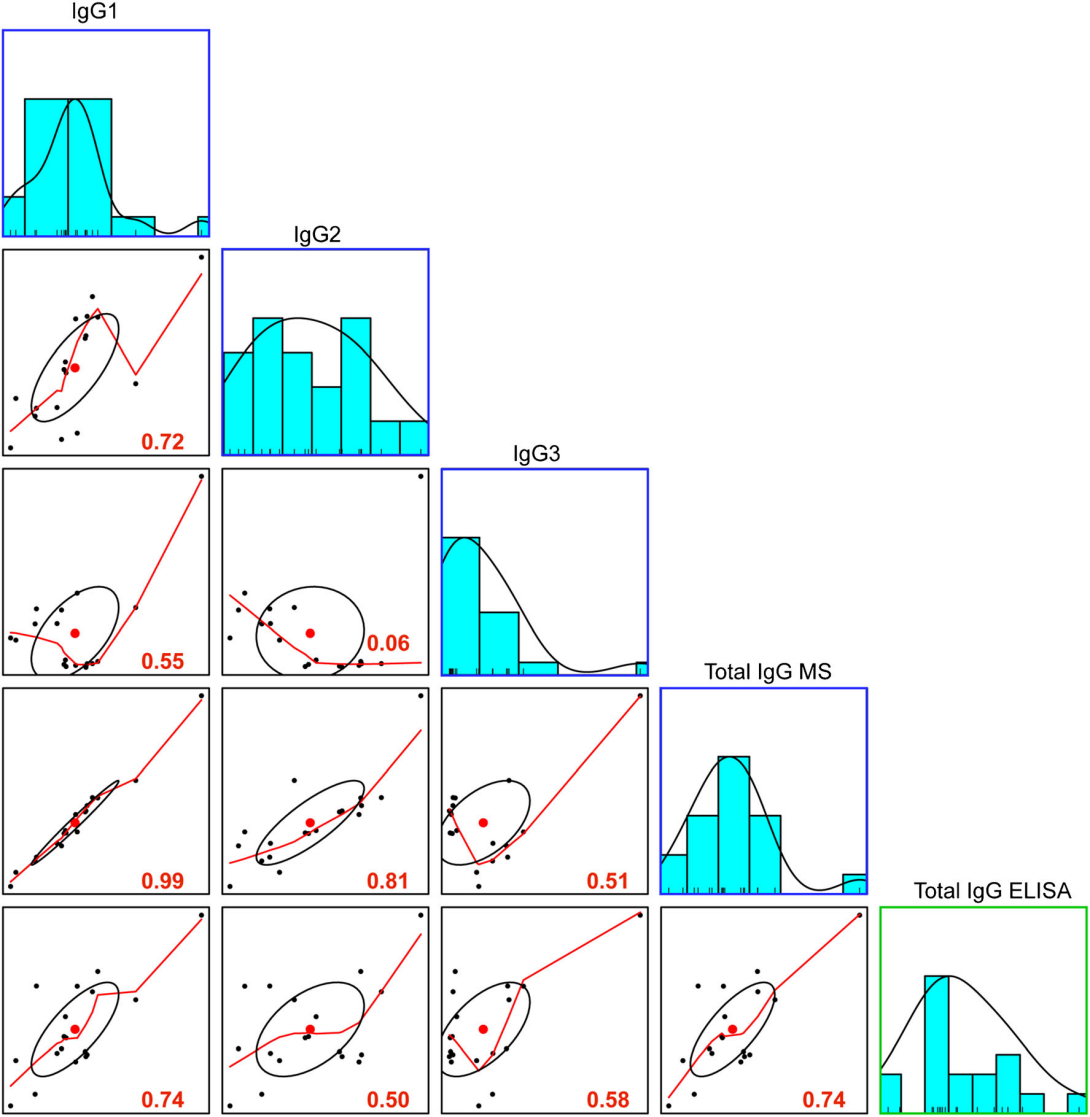
Pearson correlation of mean concentrations (μg/mL) of IgG from MS and ELISA. The distribution of the mean concentrations for immunoglobulin components, MS IgG1, IgG2, IgG3, total IgG MS, and total IgG ELISA, is shown on the diagonal. MS data is depicted in blue boxes and ELISA data in green boxes. On the bottom of the diagonal, the bivariate scatterplots with fitted lines are displayed. The Pearson correlation is indicated in the lower-right-hand corner of the bivariate scatter plot in red, where correlation values above 0.6 were considered to indicate a strong degree of correlation

Comparative MS and ELISA data indicate that for IgG, the ELISA (commercially available kit) trends are in line with MS trends over lactation (Fig. 3). Adding up all MS IgG subclass concentrations is in line with the obtained ELISA concentrations, though the MS concentrations are an underestimation relative to those from ELISA since the MS data only quantified the heavy constant parts (Fig. 3). Overall temporal trends and concentrations of IgG MS and ELISA data result in a strong Pearson correlation of 0.74 (Fig. 4). However, these same temporal and concentration trends were not observed in the MS and ELISA IgA data.

The trends observed for IgG MS and ELISA data are not observed for IgA, especially for the commercial ELISA kit, which deviates more from the MS data than the human milk–specific sIgA ELISA (Fig. 2c, d). The overall trends across lactation for both donors from MS data are more in line with the human milk–specific sIgA ELISA than the serum IgA ELISA. Moreover, there are large discrepancies in the concentration of IgA and sIgA between the two ELISA methods, Table 2. However, to make estimates of the concentration by MS relative to ELISA is more difficult, because of the overall structural complexity of sIgA. For example, even using the most ideal sample, donor one week 3, where the assumed to measured percentages of JC and SC are both 100 (Table 1), the MS data results in a sIgA concentration of approximately 600 μg/mL. With this example, we assume that all JC and SC are bound to total sIgA. However, this results in an overestimation as we cannot distinguish how much JC and SC are bound to either sIgA1 or sIgA2, the association of SC with IgM and free SC. Overall, this estimation in concentration exceeds that of both ELISA methods by > 100 μg/mL.

**Table 2:** Comparative ELISA results

	Human milk sIgA (μg/mL)			Serum kit IgA (μg/mL)		Serum kit IgG (μg/mL)	
	Mean	SD	CV (%)	Mean	SD	Mean	SD
Donor 1 Week 1	460	6	1	604	7	64	1
Donor 1 Week 2	537	24	4	704	23	95	5
Donor 1 Week 3	313	11	4	344	1	49	2
Donor 1 Week 4	235	15	6	791	19	45	5
Donor 1 Week 6	189	23	12	270	6	125	6
Donor 1 Week 8	225	1	1	344	10	69	3
Donor 1 Week 10	129	39	30	219	7	67	3
Donor 1 Week 12	283	6	2	364	6	89	1
Donor 1 Week 16	183	8	5	436	15	95	0
Donor 2 Week 1	1398	109	8	676	2	74	3
Donor 2 Week 2	870	8	1	662	4	66	2
Donor 2 Week 3	573	39	7	491	9	67	1
Donor 2 Week 4	363	6	2	259	14	65	1
Donor 2 Week 6	488	15	3	438	15	63	11
Donor 2 Week 8	701	10	1	315	6	73	4
Donor 2 Week 10	739	18	2	358	17	93	2
Donor 2 Week 12	824	25	3	455	18	82	1
Donor 2 Week 16	477	1	0	359	2	101	9

**Fig. 5:**
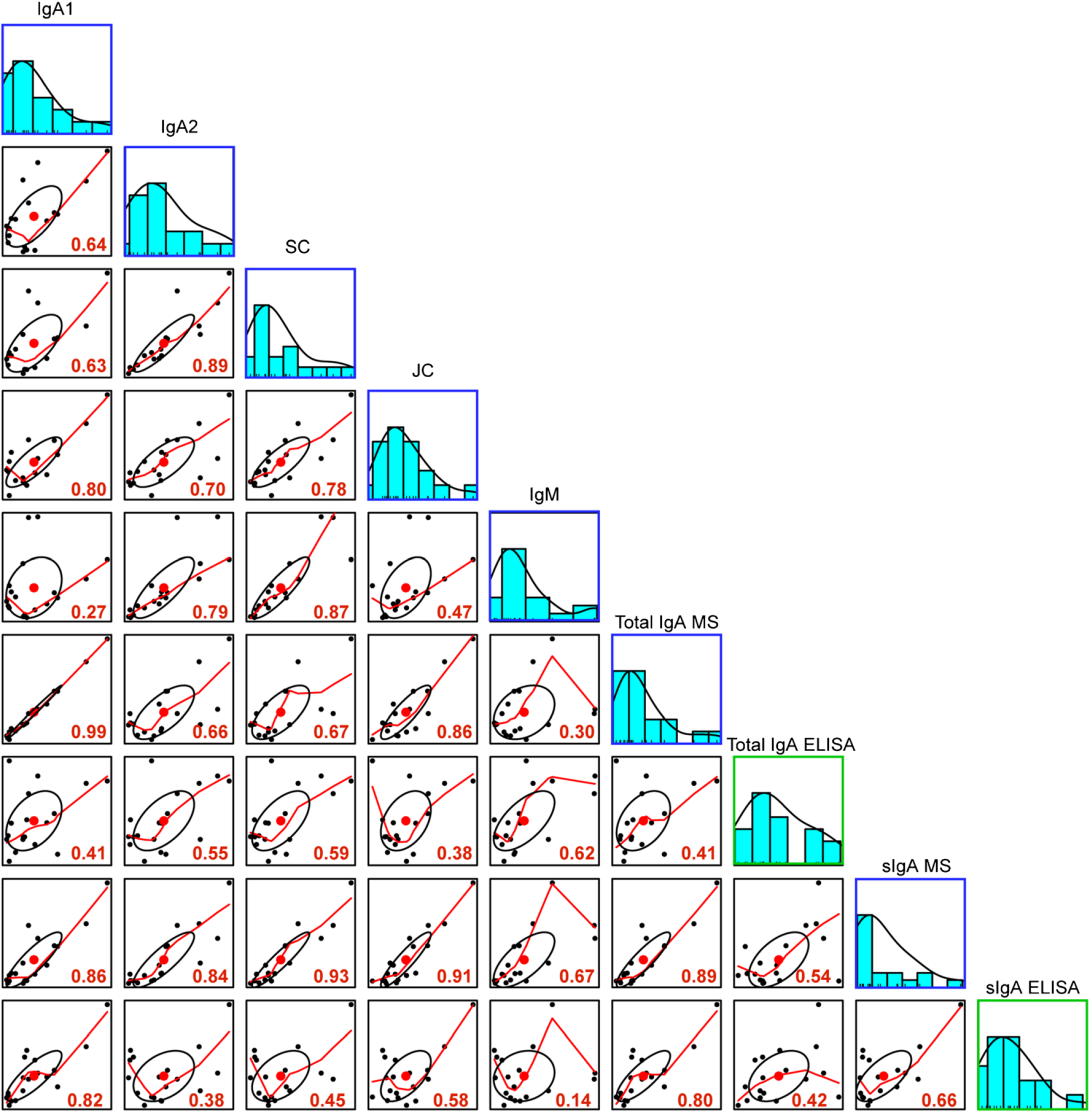
Pearson correlation of mean concentrations (μg/mL) of IgA from MS and ELISA. The distribution of the mean concentrations for immunoglobulin components, MS IgA1, IgA2, IgM, JC, SC, total IgA MS, sIgA MS, total IgA ELISA (from serum kit), and sIgA ELISA, is shown on the diagonal. MS data is depicted in blue boxes and ELISA data in green boxes. On the bottom of the diagonal, the bivariate scatterplots with fitted lines are displayed. The Pearson correlation is indicated in the lower-right-hand corner of the bivariate scatterplot in red, where correlation values above 0.6 were considered to indicate a strong degree of correlation

For this reason, looking at the individual components of IgA analyzed by MS and ELISA by Pearson correlation can provide better insights. We observe that MS-detected IgA1 very strongly correlates with the human milk–specific sIgA ELISA (r = 0.82), while MS-detected IgA2 was weakly correlated with the human milk–specific sIgA ELISA (r = 0.38); see Fig. 5. The difference in correlation of the two IgA subclasses could be due to the relatively large concentration difference in human milk, wherein IgA1 ranged in concentration from 600 to 250 μg/mL (donor two) relative to IgA2 which only reached concentrations of 10 μg/mL (donor 2). This drastic difference in overall concentration could account for the observed correlation differences with IgA1 and IgA2 MS and ELISA data. Another possible explanation is that the developed human milk–specific sIgA ELISA is better suited to assess IgA1 than IgA2, but as IgA1 accounts for the majority of the total IgA concentration in human milk, this is a suitable indicator for total sIgA.

JC and SC only had moderate correlations (r = 0.58 and 0.45, respectively); see Fig. 5. The lack of a strong correlation between JC and SC and sIgA ELISA is likely due to JC associating also with IgM, and because human milk also contains free SC. As a further confirmation that our developed human milk–specific sIgA ELISA is superior to that of the serum IgA kit, we looked for unspecific interaction with IgM. Here, the serum IgA kit was strongly correlated with IgM whereas our sIgA ELISA was very weakly correlated (r = 0.62 and 0.14, respectively) (Fig. 5).

The complexity of sIgA is further compounded by its structural components, containing both JC and SC, and being able to exist in multiple oligomeric states regardless of isotype. While sIgA1 and sIgA2 are most commonly in dimeric states, it has recently been shown that they exist as a trimer and tetramer, though these forms have been reported at mucosal surfaces, and have not been shown in biofluids [4]. However, a recent structural analysis showed that IgA2 could form pentamers similar in structure to IgM [5]; as this work was done with synthetic Fcs only, further work from human samples is needed for confirmation. In contrast, serum IgA is not thought to be secretory, so without the SC component, and to exist mostly in a monomeric state. Therefore, the variations in the structure of sIgAs between body fluids and the overall complexity of the human milk matrix together may explain the differences observed between the serum ELISA IgA and human milk–specific sIgA ELISA tests here. Additionally, the high degree of correlation of IgA2 and IgM with the serum kit could be due to the aforementioned proposed structural relatedness of IgA2 and IgM [5]. As IgM exists as a pentamer in serum and human milk, it is possible that IgA2 exists in a pentameric state as well. Human milk makes an ideal biofluid to further study the structure of IgA2 as it is found in much higher concentrations in human milk than in serum. Overall, as IgA1 is the most abundant Ig in human milk, it is not surprising then that it has the highest correlation between MS and ELISA data and therefore we can use it as our most reliable marker for assay validation.

### General trends for Igs across methods

Our developed human milk–specific sIgA ELISA allows for the assessment of longitudinal changes between different donors. Differences in Igs’ general trends are consistent between the MS- and ELISA-derived data (Figs. 2 and 3). The MS data indicate that IgA1 is the most abundant Ig throughout lactation (Fig. 2). Like IgA1, the subunits contributing to sIgA, JC and SC, have a higher concentration in early lactation and gradually decline throughout lactation (Fig. 2). The human milk–specific sIgA ELISA is in line with these MS-observed lactational trends (Fig. 2). Regardless of the assay, at any given time over the 16-week period, concentrations of sIgA1 and sIgA2 were higher in donor two than in donor one, highlighting the highly individual nature of this immunoglobulin. The most abundant IgG from the MS data is IgG1, in line with the serum IgG ELISA (Fig. 3). Overall, our results indicate that sIgA1 was the main product of maternally transferred adaptive immunity.

## Summary and future perspectives

Human milk Igs are crucial components of functional proteins as they are transported from mother to infant, providing protection when the adaptive immune system of the infant is still maturing [26]. It is known that specific classes of Igs, IgGs, are transferred from mother to fetus during pregnancy [27]. This is important as the production of endogenous IgGs in the infant develops after birth [28]. However, sIgAs are not transferred in utero [29] and are not produced by the infant until 7–30 days post parturition. Moreover, it is estimated that infants do not produce adult levels of up to 3 g/day of sIgA into the gut, until 2 years of age [30]. Therefore, human milk serves as the first source of sIgA and a continuing source throughout lactation. Increased sIgAs in early lactation and IgGs in late lactation in milk may provide a means to complement the synchronous decrease in IgG and lack of gut secretion of sIgA in infants after birth and during early infancy. Igs are highly donor specific as they carry the personal memory of pathogens faced by the individual mother [31]. Therefore, having precise, human milk–specific methods like the MS and ELISA methods is of paramount importance to investigate the specific role of Igs in early life.

Here, we have provided a validated human milk–specific sIgA ELISA method substantiated by MS data. We were able to show that relative to a serum IgA ELISA kit (moderate Pearson correlation coefficient 0.54 to MS data), our newly developed ELISA was better correlated to MS data for sIgA (strong Pearson correlation coefficient 0.66). Even with their differences, MS and ELISA technologies are complementary to one another and can therefore be used to support and validate broad and detailed human milk Ig analyses. We used MS data from human milk samples to better understand and develop an improved ELISA for the complex protein sIgA. From our data, we concluded that methods made for serum applications, like serum IgA ELISA kits, are not directly applicable to human milk. However, this appeared to be protein specific, as the tested serum IgG ELISA performed well on human milk samples compared to the respective MS data. The differences in the application of differing Ig serum ELISA kits to human milk could be due to the inherent differences in Ig structural complexity in serum and human milk, mainly that IgG and IgA in serum are monomers and in human milk sIgA has complexed to SC and exists at higher-order oligomeric states.

A limiting component to the human milk sIgA ELISA method established here is the inability to distinguish between sIgA1 and sIgA2, and that obtained data are a measure of total sIgA. For the identification of sIgA1 and sIgA2, two different new ELISAs or a multiplex-based method should be developed and validated. A strength of our assay is that we can achieve more comparable sIgA lactational trends and concentrations with our ELISA method relative to what is indicated by MS data. Even though sIgA1 and sIgA2 cannot be distinguished with the ELISA method, we confirmed by MS data that sIgA1 is the more abundant form in human milk. However, an advantage of ELISA over MS methodologies is that ELISA is more specific to functional (i.e., intact) sIgA, which cannot be determined by MS. Assessing functionality is not possible with LFQ MS data as, bottom-up methods digest intact proteins into peptide fragments, and therefore, functionality information is lost.

Future studies aiming to develop even more distinct and specific ELISA assays should investigate the effect of glycosylation and antibody binding. This is an important consideration as post-translational modifications (PTMs) are often neglected in or removed from the analysis to avoid complications. However, glycosylation is an important component of functionality for most proteins including sIgA. This could also lead to ELISA methods in which sIgA1 and sIgA2 can be distinguished from one another as they differ in glycosylation per hetero-dimer, where sIgA1 has 2 *N-*glycan and 9 *O-*glycan sites (range from 3 to 6 occupied sites), and sIgA2 has 5 *N-*glycan sites (typically 4 are reported as occupied) [32–35]. This complex secretory protein has additional glycosylation on JC, 1 *N-*glycan site, and SC has 7 *N-*glycan sites [36]. The extent of this PTM on binding affinity with ELISA methods has not been tested. However, it is known that removal of these glycans decreases the interaction of sIgA with gram-positive bacteria [37], indicating that changing the glycosylation of sIgA changes the functionality. Future work on the effects of glycosylation and ELISA development could implement the use of MS as a complementary method as glycan annotation by MS has advanced in recent years, enabling the analysis of both glycan composition and glycosite annotation in human milk [14].

## Supplementary information

ESM 1 (DOCX 829 kb)

## Data Availability

The mass spectrometry proteomics data have been deposited to the ProteomeXchange Consortium via the PRIDE [38] partner repository with the dataset identifier PXD014917 and 10.6019/PXD014917.
