# Supplementary material for: Optimization of a human milk–directed quantitative sIgA ELISA method substantiated by mass spectrometry
Source: Anal Bioanal Chem. 2021 Jun 25;413(20):5037–49. doi: 10.1007/s00216-021-03468-4 (PMC8405464; doi:10.1007/s00216-021-03468-4)
Supplement: Supplementary file 1 — (DOCX 829 kb) [file 216_2021_3468_MOESM1_ESM.docx]

**Table S1:** Determination of a 7 point standard curve

|  | **Well** | **Concentration/Dilution (ng/ml)** | **450** | **Mean OD** | **SD** | **%CV** | **∆OD** |
| --- | --- | --- | --- | --- | --- | --- | --- |
| **STD1** | A1 | 640 | 4.13 | 4.11 | 0.03 | 0.76 | 0.40 |
|  | A2 | 640 | 4.09 |  |  |  |  |
| **STD2** | B1 | 320 | 3.72 | 3.70 | 0.02 | 0.46 | 0.79 |
|  | B2 | 320 | 3.69 |  |  |  |  |
| **STD3** | C1 | 160 | 2.92 | 2.92 | 0.01 | 0.22 | 0.97 |
|  | C2 | 160 | 2.91 |  |  |  |  |
| **STD4** | D1 | 80 | 2.00 | 1.94 | 0.07 | 3.82 | 0.70 |
|  | D2 | 80 | 1.89 |  |  |  |  |
| **STD5** | E1 | 40 | 1.27 | 1.24 | 0.04 | 2.90 | 0.51 |
|  | E2 | 40 | 1.22 |  |  |  |  |
| **STD6** | F1 | 20 | 0.76 | 0.73 | 0.03 | 4.52 | 0.29 |
|  | F2 | 20 | 0.71 |  |  |  |  |
| **STD7** | G1 | 10 | 0.45 | 0.44 | 0.00 | 0.80 | 0.39 |
|  | G2 | 10 | 0.44 |  |  |  |  |
| **BLK** | H1 | 0 | 0.06 | 0.06 | 0.00 | 2.48 | 0.06 |
|  | H2 | 0 | 0.06 |  |  |  |  |

**Table S2:** Sample concentration variability over freeze thaw cycles

|  | | **1x freeze thaw** | **2x freeze thaw** | **3x freeze thaw** | **Overall** |
| --- | --- | --- | --- | --- | --- |
| **Sample 1** | Mean (ug/mL) | 651.11 | 424.23 | 163.74 | 651.11 |
|  | Stdev | 78.22 | 6.01 | 36.72 | 78.22 |
|  | CV% | 12.01 | 1.42 | 22.43 | 8.77 |
| **Sample 2** | Mean (ug/mL) | 940.84 | 681.61 | 331.57 | 940.84 |
|  | Stdev | 43.64 | 33.72 | 4.59 | 43.64 |
|  | CV% | 4.64 | 4.95 | 1.39 | 3.12 |

**Table S3:** Determination of assay variability for human milk concentrations

| **Sample**  **Name** | **Well** | **Dilution** | **OD** | **Concentration (ng/ml)** | **Concentration x Dilution (ug/ml)** |
| --- | --- | --- | --- | --- | --- |
| **Sample 1** | A3 | 2500 | 1.92 | 75.44 | 188.61 |
|  | A4 | 2500 | 1.87 | 72.54 | 181.34 |
|  | B3 | 5000 | 1.18 | 38.77 | 193.83 |
|  | B4 | 5000 | 1.13 | 36.81 | 184.06 |
|  | C3 | 10000 | 0.66 | 17.76 | 177.61 |
|  | C4 | 10000 | 0.63 | 16.54 | 165.37 |
|  | D3 | 20000 | 0.34* | 4.29 | 85.85 |
|  | D4 | 20000 | 0.39* | 6.66 | 133.22 |
| **Sample 2** | A5 | 2500 | 2.67 | 132.72 | 331.79 |
|  | A6 | 2500 | 2.66 | 132.12 | 330.30 |
|  | B5 | 5000 | 1.74 | 65.22 | 326.09 |
|  | B6 | 5000 | 1.76 | 66.40 | 332.02 |
|  | C5 | 10000 | 1.03 | 32.56 | 325.63 |
|  | C6 | 10000 | 1.04 | 33.10 | 330.99 |
|  | D5 | 20000 | 0.64 | 16.93 | 338.65 |
|  | D6 | 20000 | 0.64 | 16.85 | 337.07 |

*Values outside of acceptable range

**Table S4:** Spike protein recovery

|  | **Dilution** | **Spike amount** | **concentration** | **Concentration + spike** | **Spike recovery** | **Recovery**  **(%)** |
| --- | --- | --- | --- | --- | --- | --- |
| **Sample 1** | 2500 | 80 | 73.99 | 134.81 | 60.82 | 76.03 |
|  | 5000 | 80 | 37.79 | 107.37 | 69.58 | 86.97 |
|  | 10000 | 80 | 17.15 | 90.92 | 73.77 | 92.21 |
|  | 20000 | 80 | 5.48 | 89.37 | 83.90 | 104.87 |
| **Sample 2** | 2500 | 80 | 132.42 | 180.91 | 48.49 | 60.61 |
|  | 5000 | 80 | 65.81 | 132.23 | 66.42 | 83.02 |
|  | 10000 | 80 | 32.83 | 104.28 | 71.45 | 89.31 |
|  | 20000 | 80 | 16.89 | 90.49 | 73.59 | 91.99 |

**
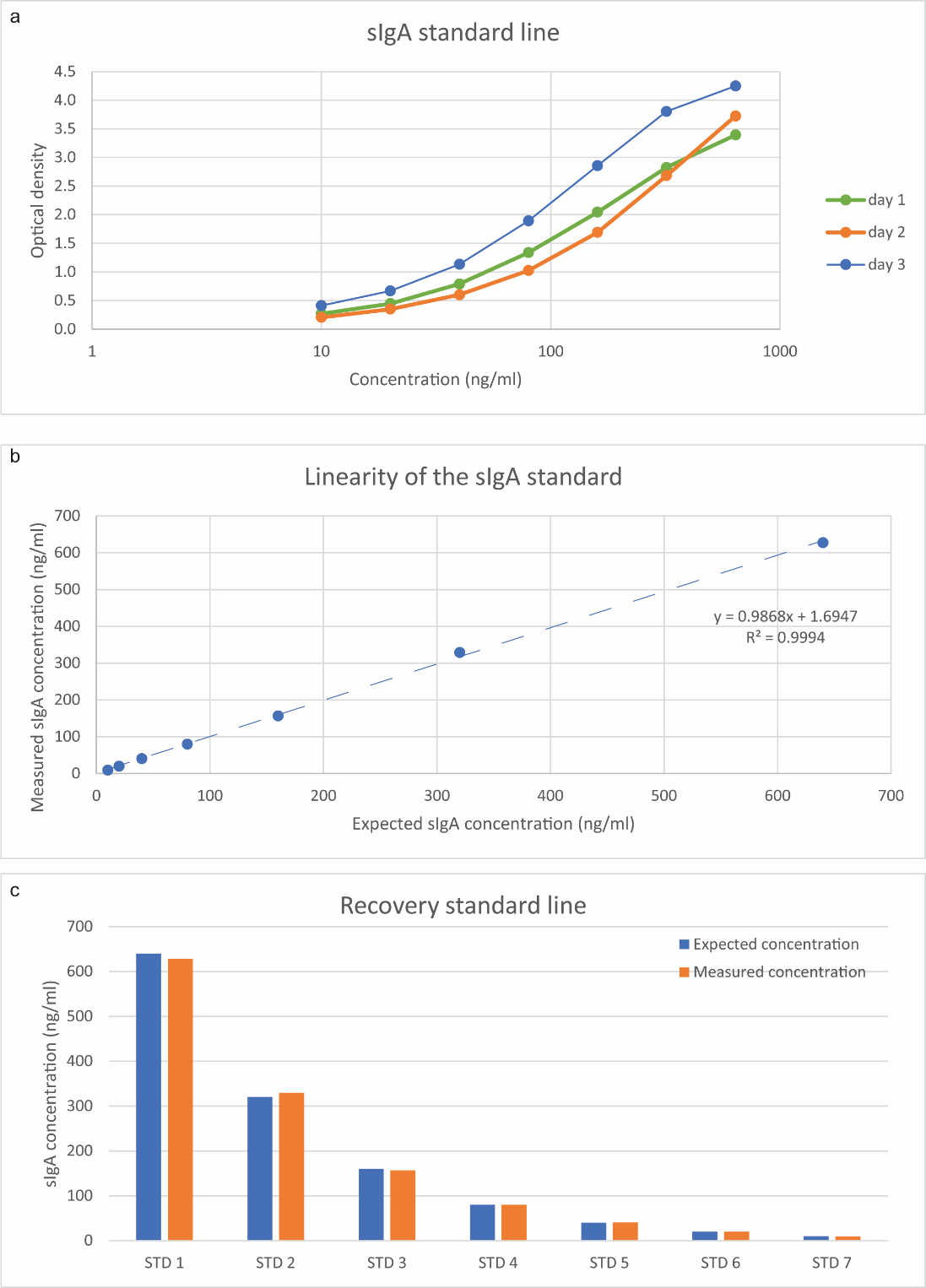
**

**Fig S1 Assessing the concentration range, linearity and recovery of the sIgA standard.** a) The concentration range of the sIgA standard is plotted as optical density (OD) by concentration (ng/mL) and was measured across 3 different days. The average %CV of the OD within each assay day was below 15%, across the 3 days %CV ranged from 11-36% indicating that for best results assays should be performed on the same day. b) The linearity of measured vs expected sIgA concentration of the standard curve across 3 days is depicted. The concentration of the standard curve remained stable across the 3 days with a high R^2^ values. c) The recovery of measured vs expected sIgA concertation is depicted as bars for each of the 7 standards in the curve. Recoveries ranged from 94-103%. Values for the 7 point standard curve are provided in Table S1.

**
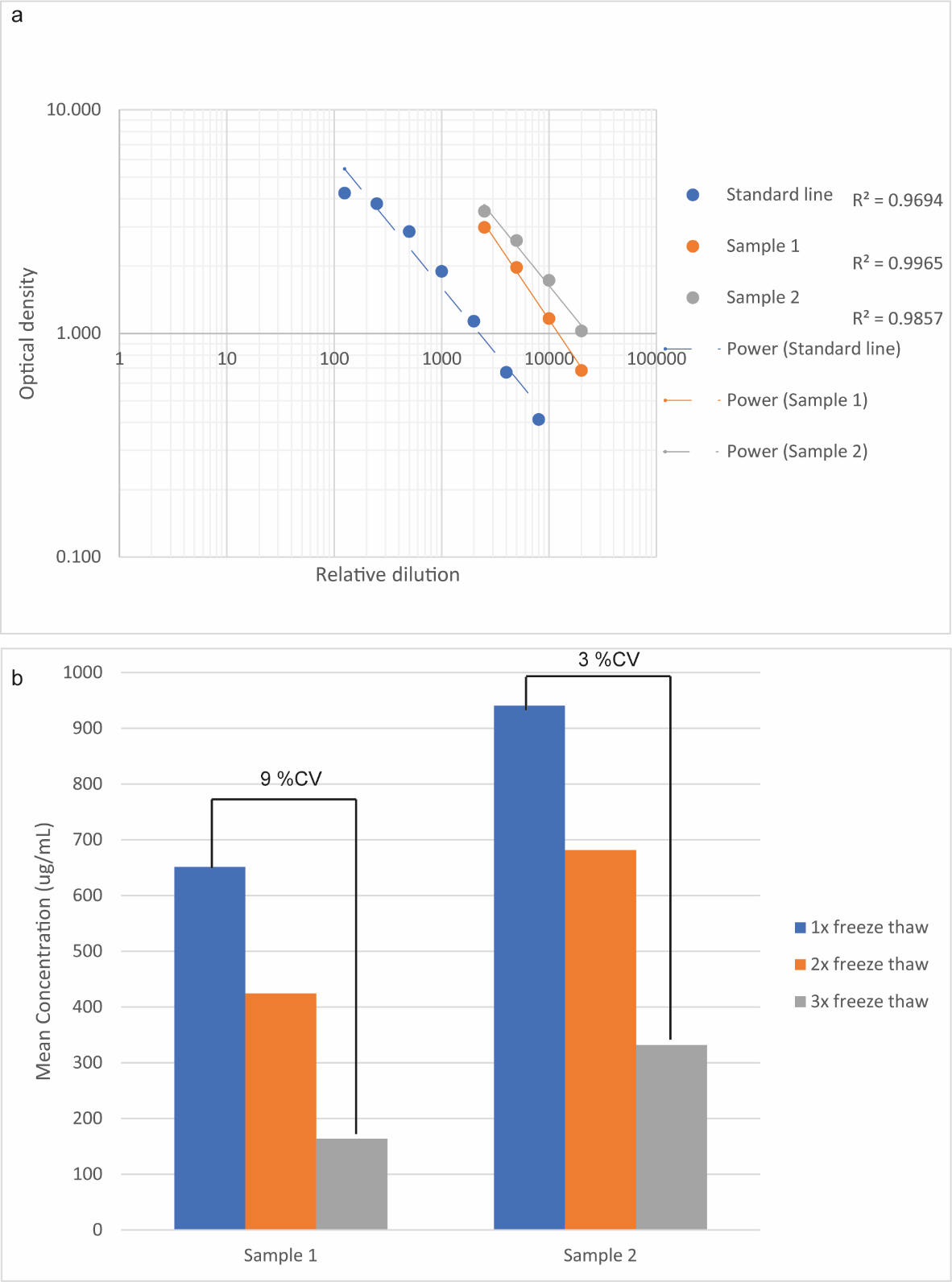
**

**Fig S2 Sample linearity human milk relative to the standard curve and variability across multiple freeze thaw cycles.** a) The linearity of the standard curve with a dilution range of 125-8000 is plotted against two human milk samples. Both human milk samples were within the linear range of the standard curve with high R^2^ values, indicating the working range of the sIgA ELISA assay. b) The mean concentration of ug/mL is plotted for two human milk samples across three freeze thaw cycles. For both samples 3 freeze thaw cycles was still within the acceptable range of a %CV < 25. Values are for the bar chart are provided in Table S2.

**
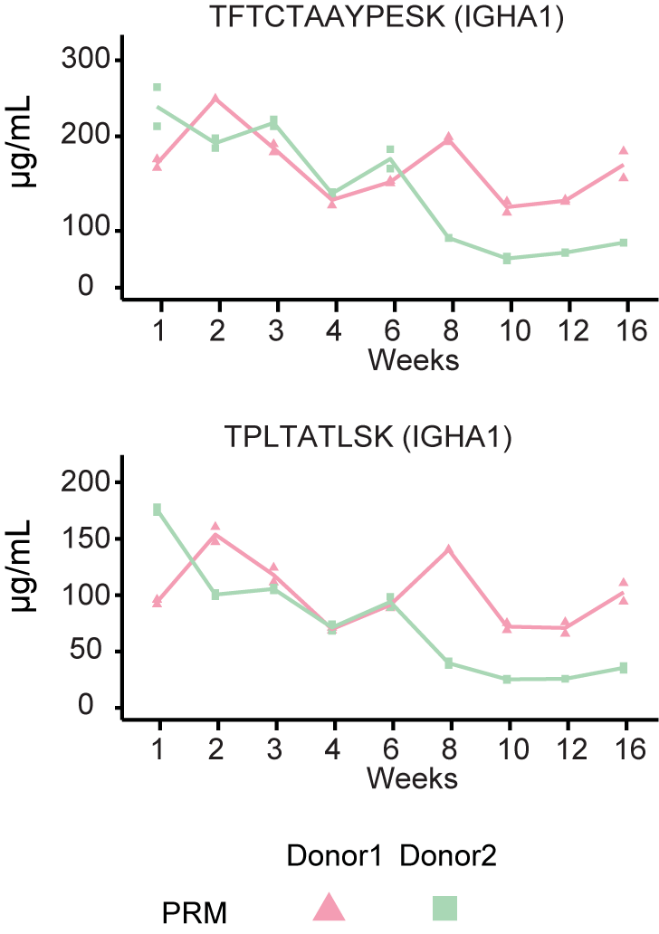
**

**Fig S3 Validation of the abundance of IgA1 by targeted proteomics.**

Trends in abundance of IgA1 was monitored by PRM using two peptides. Trends and concentration for IgA1 followed LFQ trends in Fig 2. Data points indicate the values of each technical replicate, lines are linked by the median of the data points in each week for each donor.
